# Florence Nightingale

**Published:** 1910-09

**Authors:** 


BUFFALO MEDICAL JOURNAL

A Monthly Review of Medicine and Surgery

*EDITOR*

WILLIAM WARREN POTTER, M. D.

All communications, whether of a literary or business nature, books for review and xohangcs, should be addressed to the editor, 238 DELAWARE AVENUE, BUFFALO, N.Y.

VOL. LXVI. SEPTEMBER, 1910. No. 2

### Florence Nightingale

THE most distinguished woman of her time, Miss Florence Nightingale, died in London, August 13, 1910, aged ninety years. For fifty-five years her name had been familiar to the public, not only as that of the first woman to enter service as an army nurse but as one to mitigate the suffering of the soldiers in the field and to contribute materially toward the reduction of the death rate in the Crimea. The writer well remembers that in the winter of 1855, the newspaper press recorded the reception given Miss Nightingale on her arrival at the seat of war, and the good work she accomplished from time to time thereafter.

In this sketch of her life, which we publish as an example to be emulated, the material has been compiled from various sources. She was born in 1820 in Florence, from which she received her name, being the younger daughter of a Sheffield banker named Shone, who adopted the name of Nightingale in accordance with the terms of the will of one Peter Nightingale, whose property fell to him.

For some years before the beginning of the Crimean war, she had taken up what was to become her life work, and during a period of thirteen years had devoted her attention and energy to the organisation and improvement of hospitals. She had visited all the hospitals of London, Edinburgh and Dublin; all the hospitals of Paris, where she studied with the Sisters of Charity; at Kaiserwerth, on the Rhine, where she was twice in training as a nurse; the hospitals at Berlin and others in Germany; at Lyons, Rome, Brussels, Constantinople and Alexandria, and the war hospitals of the French and Sardinians. Surely, this was a preparation that should fit her adequately for the strenuous duties about to be assumed. Let it be remembered that she chose her career in face of the fact that she had been born to wealth and influence, and had received every advantage which money and education could supply.

The opening of the Crimean War found Florence Nightingale in charge of a home for infirm and invalid governesses in London, but in 1854, the situation in the East having become intolerable, she wrote to the government, offering her services, and saying that she thought the soldiers needed good nursing such as only women could give them. At the same time a letter was on its way to her from Sir Sidney Herbert, of the War Department, who, with the feeling that it was "a woman's work, and there is one woman in England who can set this right," wrote and asked Miss Nightingale to go to the East and organise a nursing service in the great hospital at Scutari.

HORRORS AT SCUTARI.

At that time the death rate at Scutari was 42 per cent., while in the Kululi Hospital it rose to 52 per cent. The troops remained inactive, decimated by cholera and other diseases. There were more than thirteen thousand men sick in the hospitals. Four patients out of every five who underwent hospital amputation died from gangrene.

This was the situation that confronted England. The nation was kindled to a passion of mingled wrath and pity. More than $5,000,000 was poured into various relief funds, medical supplies were sent out by the ton, and the medical staff was multiplied until there was a doctor to every one hundred soldiers.

Within a week Miss Nightingale and a party of nearly fifty women started for the Crimea, where they arrived on the night before the battle of Inkerman. From then on she ruled Scutari. She developed a remarkable executive ability little dreamed of even by herself; and still more wonderful, her orders were obeyed by soldiers who never before took orders from a woman.

It is related that soon after her arrival she discovered that some much needed supplies were locked up by the commissaries, on the ground that they had not been inspected. Miss Nightingale went to the magazines, told the sergeant of the guard who she was, and asked if he would take an order from her. He replied that he would, whereupon she told him to drive in the door. When he complied the stores were distributed without further delay to the sick men who were languishing for them.

DEATH RATE REDUCED AT SCUTARI.

It was through such unusual methods as these, combined with a complete reorganisation of the system of treating the sick and wounded, that the death rate at Scutari was reduced to 2 per cent. Naturally the patients loved the woman who had directed it all. They reached forth their hands to touch the hem of her garments as she passed. Those who lacked the strength kissed her shadow as it crossed their pillows. At night, when all was still, she made the rounds with a little nurse's lamp in her hands to see that everything was in proper shape.

Florence Nightingale did not quit her post of duty beside the Bosporus until peace was declared. Then, traveling quietly and under an assumed name to avoid publicity, she reached England before it was known she had left Turkey. Try as she might she could not wholly avoid the proofs of the nation's appreciation of her worth and work. She was commanded to visit Balmoral and was thanked by the Queen in person. Lords and commons thanked her and the newspapers devoted columns on columns of space to sounding her praises.

GIFT OF A QUARTER MILLION.

A fund of £50,000, to which all classes contributed eagerly—each soldier in the army giving a day's pay—was raised by the women of England and presented to her. This testimonial she accepted, and then proceeded to spend every shilling for the establishment and maintenance of an institution for the training of nurses and hospital attendants. The result was the Florence Nightingale School for Nurses, in London, and for many years she personally directed the policy of the institution.

Many anecdotes are told of this great woman, none of which is without truthful interest. One deserves record here. A dinner given a few months after the end of the fighting, in honor of the officers who had distinguished themselves, the host, Lord Stratford, arose and announced that he would like to have an expression of opinion as to who became most distinguished in the Crimea. Recognising that some might feel a delicacy in expressing their opinion publicly, he had slips of paper distributed and each was asked to write his choice. When all had been returned and examined Lord Stratford found that the vote had been unanimous.

The name which appeared on the slips was not that of a statesman or general; it was not even the name of a man. On each slip was the name of Florence Nightingale, a recognition of the womanly tact and sympathy which had solved a problem frequently considered greater than the taking of Sebastopol.

HONORED BY THE KING.

King Edward less than four years ago, when she had reached the age of eighty-seven, conferred upon Miss Nightingale, the high honor of membership in the Order of Merit, the most exclusive distinction in the gift of the British sovereign, no woman having received the honor before. Membership in the order is restricted to twenty-four, and it has included such men as Lord Roberts, Lord Wolseley, Field Marshal Lord Kitchener, James Bryce, Prince Yamagata, Marquis Oyama, Admiral Togo, Sir George Stuart White, Lords Rayleigh, Kelvin and Lister, the

Right Honorable John Morley, Admirals of the Fleet Sir Edward Hobart Seymour and Sir John Fisher, Sir William Huggins, Sir Laurence Alma-Tadema, George Meredith, William Holman-Hunt and the Earl of Romer.

Her funeral was as quiet as possible, in accordance with her wishes, her executors having declined interment in Westminster Abbey. During recent years, owing to her feebleness and advanced age, Miss Nightingale had received few visitors. On May 12, 1910, she celebrated her ninetieth birthday, and received a congratulatory message from King George.

